# Prophylactic Ozone Administration Reduces Intestinal Mucosa Injury Induced by Intestinal Ischemia-Reperfusion in the Rat

**DOI:** 10.1155/2015/792016

**Published:** 2015-06-16

**Authors:** Ozkan Onal, Fahri Yetisir, A. Ebru Salman Sarer, N. Dilara Zeybek, C. Oztug Onal, Banu Yurekli, H. Tugrul Celik, Ayse Sirma, Mehmet Kılıc

**Affiliations:** ^1^Department of Anesthesiology and Reanimation, Selçuk University Medical Faculty, 42100 Konya, Turkey; ^2^Department of General Surgery, Atatürk Education and Research Hospital, 06600 Ankara, Turkey; ^3^Department of Anesthesiology and Reanimation, Atatürk Education and Research Hospital, 06600 Ankara, Turkey; ^4^Department of Histology and Embryology, Hacettepe University Faculty of Medicine, 06012 Ankara, Turkey; ^5^Ankara 4th Tuberculosis Dispensary, 06021 Ankara, Turkey; ^6^Department of Endocrinology, Bozyaka Education and Research Hospital, 35022 İzmir, Turkey; ^7^Department of Biochemistry, Turgut Özal University Medical Faculty, 06010 Ankara, Turkey

## Abstract

*Objectives*. Intestinal ischemia-reperfusion injury is associated with mucosal damage and has a high rate of mortality. Various beneficial effects of ozone have been shown. The aim of the present study was to show the effects of ozone in ischemia reperfusion model in intestine. *Material and Method*. Twenty eight Wistar rats were randomized into four groups with seven rats in each group. Control group was administered serum physiologic (SF) intraperitoneally (ip) for five days. Ozone group was administered 1 mg/kg ozone ip for five days. Ischemia Reperfusion (IR) group underwent superior mesenteric artery occlusion for one hour and then reperfusion for two hours. Ozone + IR group was administered 1 mg/kg ozone ip for five days and at sixth day IR model was applied. Rats were anesthetized with ketamine∖xyzlazine and their intracardiac blood was drawn completely and they were sacrificed. Intestinal tissue samples were examined under light microscope. Levels of superoxide dismutase (SOD), catalase (CAT), glutathioneperoxidase (GSH-Px), malondyaldehide (MDA), and protein carbonyl (PCO) were analyzed in tissue samples. Total oxidant status (TOS), and total antioxidant capacity (TAC) were analyzed in blood samples. Data were evaluated statistically by Kruskal Wallis test. *Results*. In the ozone administered group, degree of intestinal injury was not different from the control group. IR caused an increase in intestinal injury score. The intestinal epithelium maintained its integrity and decrease in intestinal injury score was detected in Ozone + IR group. SOD, GSH-Px, and CAT values were high in ozone group and low in IR. TOS parameter was highest in the IR group and the TAC parameter was highest in the ozone group and lowest in the IR group. *Conclusion*. In the present study, IR model caused an increase in intestinal injury.In the present study, ozone administration had an effect improving IR associated tissue injury. In the present study, ozone therapy prevented intestine from ischemia reperfusion injury. It is thought that the therapeutic effect of ozone is associated with increase in antioxidant enzymes and protection of cells from oxidation and inflammation.

## 1. Introduction

Ozone is both strong antioxidant and oxidant agent. Ozone can oxidize all microorganisms and their toxins at different rates [1]. Studies show the benefits of ozone in cerebrovascular ischemia [2], chronic ulcer [3], arteriosclerosis obliterans [4], immune deficiency [5], hepatic steatosis [6], and heart ischemia [7]. Ozone (O_3_) is used for therapeutic purposes in the form of a gas mixture (O_2_ + O_3_), and it is found at a rate of around 3% in this mixture [8]. It has been shown that ozone administration stimulates oxidative preconditioning or enhances adaptation to oxidative stress [9]. Oxidative preconditioning exerts its protective effect by stimulating the endogen antioxidant system and decreasing glycogen consumption and lactate production. Although ozone treatment has no known harmful effects, in those with uncontrolled hyperthyroidism, sepsis, fauvism, severe blood loss and hemophilia, clotting disorders, recent myocard infarctus, and brain stroke with active bleeding and in pregnant women in the first trimester, it should not be employed unless it is absolutely necessary.

Intestinal ischemia-reperfusion (IR) is a serious condition that can emerge after hemorrhage, trauma, septic shock, severe burns, small bowel transplantation, abdominal aorta surgery, and cardiopulmonary bypass [10]. Although the reperfusion of ischemic tissue is an important repair mechanism, it has also been shown to increase acute ischemic injury via reactive oxygen and nitrogen products. Various methods have been tried to decrease IR injury in intestinal mucosa and to accelerate the regeneration of mucosal functions [11]. Among these, radical scavengers [12, 13], xanthine oxidase inhibitors, neutrophil consumers [14, 15], PAF antagonists [16], and intraluminal oxygenation can be cited. Preconditioning with ozone in IR injury was studied in skeletal muscles, renal tissues, and brain. In this study the effect of ozone preconditioning was investigated on intestinal IR injury model.

## 2. Materials and Methods

The current study was approved by the local animal ethics committee and was performed in accordance with the National Institutes of Health guidelines for the use of experimental animals. Male Wistar rats weighing 250–300 g were used in this study. All rats were housed at a temperature of 24 ± 3°C with a 12-hour light-dark cycle and acclimated for seven days before study. The animals were fed with a standard pellet diet and water ad libitum.

### 2.1. Experimental Groups

Animals were randomly divided into four groups with seven rats in each group. The number of rats in the groups was determined according to the previous studies [17, 18] and limited to twenty-eight in order to eliminate the excessive sacrification. The control group was given serum physiologic (SF) intraperitoneally (ip) for five days and at the sixth day IR injury was applied. The ozone group was given ozone/oxygen mixture at a dosage of 1 mg/kg single intraperitoneal dose per day for five days and IR injury was not applied. The ischemia reperfusion model was performed for ischemia reperfusion (IR) group and SF or ozone was not given. Ozone + IR group was administered at 1 mg/kg ip ozone for five days before applying the ischemia reperfusion model and IR injury was performed at sixth day.

### 2.2. Ozone Preconditioning

Ozone was generated by ozone generator machine (Evozone Basic Plus, Reutlinger, Germany). Ozone generator machine controls the gas flow rate and ozone concentration in real time by a built-in UV spectrometer. The ozone flow rate was kept constant at 3 L/min gas mixture of 97% O_2_ + 3% O_3_, representing a concentration of 60 *μ*g/mL and the volume of gaseous mixture administered ip to each animal was approximately 3.2–4.2 mL. Tygon polymer tubes and single-use silicon-treated polypropylene syringes (ozone resistant) were used throughout the reaction to ensure containment of O_3_ and consistency of concentrations.

The first dose of ozone was administered 6 days before ischemia. In total 5 doses of ozone were administered. Duration of ozone administration was also determined in view of previous studies. According to the study of Merin et al. ozone was administered during seven days before ischemia [17], three days four-dose in the study of Ekici et al. [18], and in the study of Koca et al. [19], overall 4-dose was administered which was sufficient. Taking these studies into consideration, five doses of ozone were administrated.

### 2.3. The Ischemia Reperfusion Model

A standard model of ischemia reperfusion was used as described previouslyin our study [20]. The rats were anesthetized with ketamine 80 mg/kg ip and xzylazine 5 mg/kg ip before ischemia reperfusion and supplementary doses of ketamine xzylazine were given at appropriate intervals during ischemia and reperfusion period. The rats were allowed to breathe spontaneously. Superior mesenteric artery (SMA) was isolated near its aortic origin via midline laparotomy in all animals. Both this artery and collateral branches coming from the celiac axis and inferior mesenteric artery were occluded with a traumatic vascular clip for 60 minutes. The absence of arterial pulsation distal to the clip or pale color of small intestine confirmed adequate occlusion. Entire bowel was covered with sterile pads soaked in saline at 37°C in order to lessen heat loss and evaporation. After 1 hour the clip was removed and the intestine was allowed to reperfuse for 2 hours. The return of pulses and recoloration of small intestine were assumed to indicate adequate reperfusion of intestine.

### 2.4. Tissue Sampling

On the sixth day, at the end of the IR period (1 h. ischemia and 2 h. reperfusion), the animals were sacrificed, and tissue samples were obtained from ileum and jejunum for histopathological examination and superoxide dismutase (SOD), glutathione peroxidase (GSH-Px), catalase (CAT), malondialdehyde (MDA), and protein carbonyl (PCO) analysis. Blood samples were also collected for total oxidant score (TOS), and total antioxidant capacity (TAC) analysis.

### 2.5. Histopathological Examination

Intestinal samples from the proximal jejunum and distal ileum were fixed with 10% formalin. After tissue processing, all segments were embedded in paraffin wax. The tissues were sectioned with a 5 *μ*m thickness and stained with hematoxylin and eosin stain.

The specimens were examined and photographed under a light microscope with a DC490 digital camera (Leica, Wetzlar, Germany) by two histologists who were blinded to the study. The villus height and crypt depth for each specimen were measured in 10 villi and 10 crypts in the jejunum and ileum sections using the Leica Application Suite image analysis software (Leica, Wetzlar, Germany). Five random fields were examined under 20x objective by two investigators and injury in the intestinal mucosal tissues in all groups was graded semiquantitatively according to Chiu's classification [21]: 0, normal mucosal villi; 1, subepithelial space at the tips of the villi; 2, moderate elevation of the epithelial layer from the lamina propria; 3, massive epithelial elevation extending down the sides of the villi (a few tips may be denuded); 4, denuded villi with the lamina propria exposed and dilated capillaries; and 5, disintegration of the lamina propria, haemorrhage, and ulceration.

### 2.6. Biochemical Analysis

For the biochemical analysis, tissues were washed two times with cold saline solution, placed into glass bottles, and stored in a deep freezer at −80°C until processing. The frozen intestinal tissues were homogenized in phosphate buffer (pH 7.4) by means of a homogenizator (Ultra Turrax IKA T18 Basic, IKA Labortecnic, Staufen, Germany) in an ice cube. The homogenates were centrifuged at 14,000 rpm (7,530 g) at 4°C for 10 min, and the supernatant was analyzed. MDA, CAT, GSH-Px, SOD, and PCO were measured in intestine tissue samples.

TOS and TAC were measured in the blood samples. For these analyses, the blood samples were centrifuged at 3,500 rpm for 15 min and the serum was collected and stored at −80°C until processing.

### 2.7. Measurement of Malondialdehyde (MDA)

The lipid peroxidation product and intestinal tissues were homogenized in 1.15% KCl solution. A 100 *μ*L aliquot of the homogenate was added to a reaction mixture containing 200 *μ*L 8.1% sodium dodecyl sulfate, 1.500 *μ*L 20% acetic acid (pH 3.5), 1.500 *μ*L 0.8% thiobarbituric acid, and 700 *μ*L distilled water. Samples were then boiled for 1 h at 95°C and centrifuged at 3.000 g for 10 min. The absorbance of supernatant was measured by spectrophotometry at 650 nm.

### 2.8. Measurement of Catalase (CAT)

Cayman's catalase assay kit was used to determine the activity of catalase. The method is based on the reaction of the enzyme with methanol in the presence of an optimal concentration of H_2_O_2_. The formaldehyde produced was measured calorimetrically in tissue homogenate.

### 2.9. Measurement of Glutathione Peroxide (GSH-Px)

Cayman GSH-Px assay kit was used to measure the activity of GSH-Px. The rate of decrease in the A 340 is directly proportional to the GSH-Px activity in tissue sample.

### 2.10. Measurement of Superoxide Dismutase (SOD)

Superoxide dismutase activity was evaluated by inhibition of nitro blue tetrazolium reduction by superoxide anion generated by the xhanthine/xhanthineoxide system using a commercial assay kit (Nanjing Jiancheng Biological Product, Nanjing China.)

### 2.11. Measurement of Protein Carbonyl (PCO)

Cayman's protein carbonyl colorimetric assay kit was used. The amount of protein hydrozone is quantified spectrophotometrically at an absorbance between 360 and 385 nm.

### 2.12. Measurement of Total Oxidant Status (TOS)

TOS levels of blood samples were determined by using an automated colorimetric measurement method [22]. It was performed by using an Aeroset 2.0 analyzer and a commercial Cayman's TOS kit. The assay is based on the oxidation of ferrous ion to ferric ion in the presence of various oxidant species in acidic medium and the measurement of the ferric ion by xylenol orange. The color intensity, which can be measured spectrophotometrically, is related to the total quantity of oxidant molecules in the sample and the assay is calibrated with hydrogen peroxide [22].

### 2.13. Measurement of Total Antioxidant Capacity (TAC)

Measurement of TAC was performed using an aero set 2.0 analyzer and a Cayman's total antioxidant score kit by using a novel automated measurement method [23]. In this method, the hydroxyl radical, the most potent radical, is produced via Fenton reaction and consequently the colored dianisidine radical cations, which are also potent radicals, are produced in the reaction medium of the assay. Antioxidant capacity of the added sample against these colored potent free radical reactions measures the total antioxidant capacity. The reaction rate is calibrated with Trolox, which is widely used as a traditional standard for TAC measurement assays.

### 2.14. Statistical Analysis

Data analysis was performed by using SPSS for Windows, version 11.5 (SPSS Inc., Chicago, IL, United States). Whether the distributions of continuous variables were normally or not was determined by Shapiro Wilk test. Levene test was used for the evaluation of homogeneity of variances. Data were shown as mean ± standard deviation or median (IQR), where applicable. While, the differences among groups were compared by One-Way ANOVA, otherwise, Kruskal Wallis test was applied for comparisons of the median values. When the *p* value from One-Way ANOVA or Kruskal Wallis test statistics are statistically significant post hoc Tukey HSD or Conover's nonparametric multiple comparison tests, respectively, were used to know which group differs from others. A *p* value less than 0.05 was considered statistically significant.

## 3. Results

**Figure 1:**
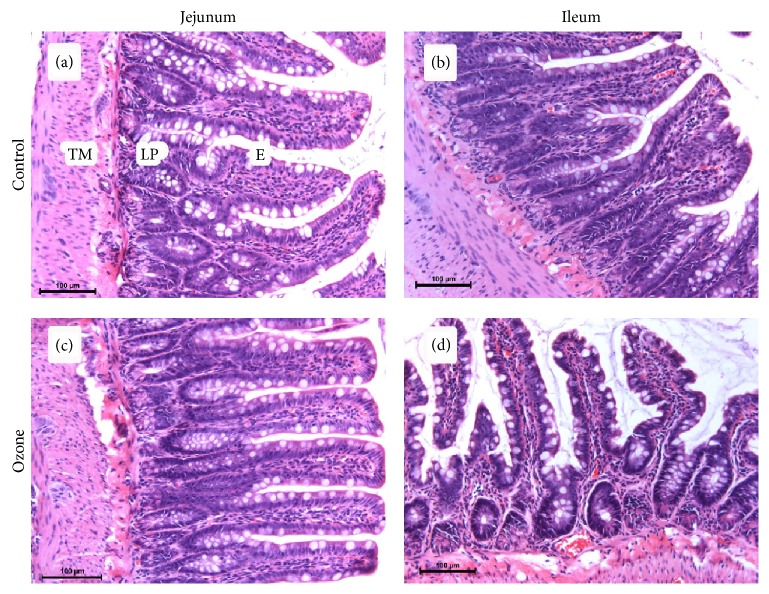
Effect of ozone in jejunum and ileum (stained with hematoxylin-eosin, magnification ×200). Normal intestinal histology of jejunum and ileum in both control (a, b) and ozone groups (c, d). TM: tunica muscularis, LP: lamina propria, E: enterocyte.

Light microscopic observation of intestine tissues revealed normal morphology with intact villi in the ozone administrated group. Simple columnar epithelium with goblet cells was observed around the villi and in crypts in both the control and ozone groups (Figure 1). There was no statistical difference in the height of the villi, the depth of the crypts and intestinal damage scores in the ozone group compared to control group (Figure 1) (Table 1).

**Figure 2:**
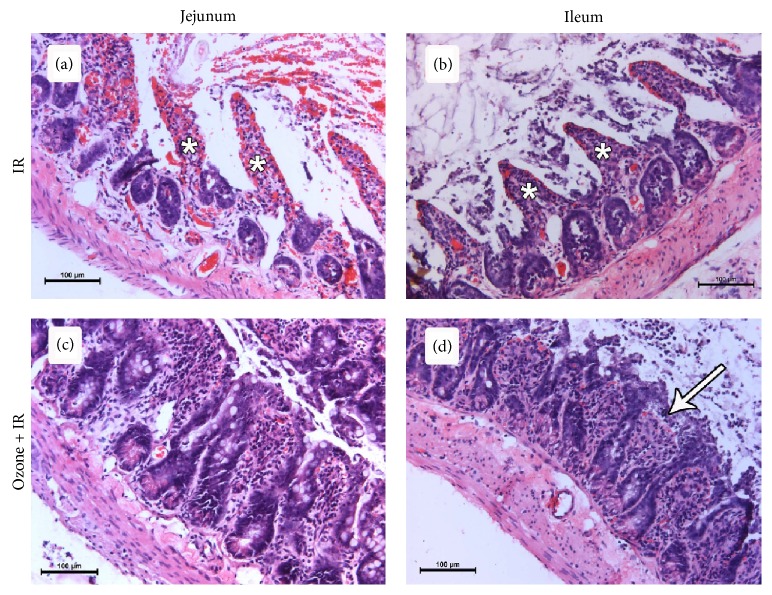
Effect of ischemia reperfusion and ozone pretreatment to ischemia reperfusion in jejunum and ileum (stained with hematoxylin-eosin, magnification ×200). Desquamation of the epithelium and denuded villi (asteriks) in both jejenum (a) and ileum (b) of IR group. Shortened and thick villi in both jejenum (c) and ileum (d) of ozone pretreated IR group. A less marked subepithelial space (arrow) at the villus tip in ileum.

The intestinal epithelium overlying the villi were lost at the tips of the villi, and in scattered places, villi epithelium was completely lost, leaving the villi denuded in both the jejunum and the ileum in the IR group (Figure 2). The height of the villi and the depth of the crypts were significantly decreased in the IR group in both the jejunum and the ileum compared to the control group (*p* < 0.001) (Table 1). The mean intestinal injury grade of the IR group was significantly increased in the jejunum and the ileum compared to the control group (Table 2).

In the group in which ozone was administered before the IR procedure, both the height of the villi and depth of the crypts were significantly increased in the jejunum in comparison to the IR group (*p* < 0.001) (Table 1). Pretreatment with peritoneal ozone prevented intestinal mucosal injury caused by IR. The villi were covered with the epithelium, maintaining their integrity, and the mean intestinal injury grade of the IR + ozone group was significantly lower in both the jejunum and the ileum compared to the IR group (Figure 2) (Table 2).

**Figure 3:**
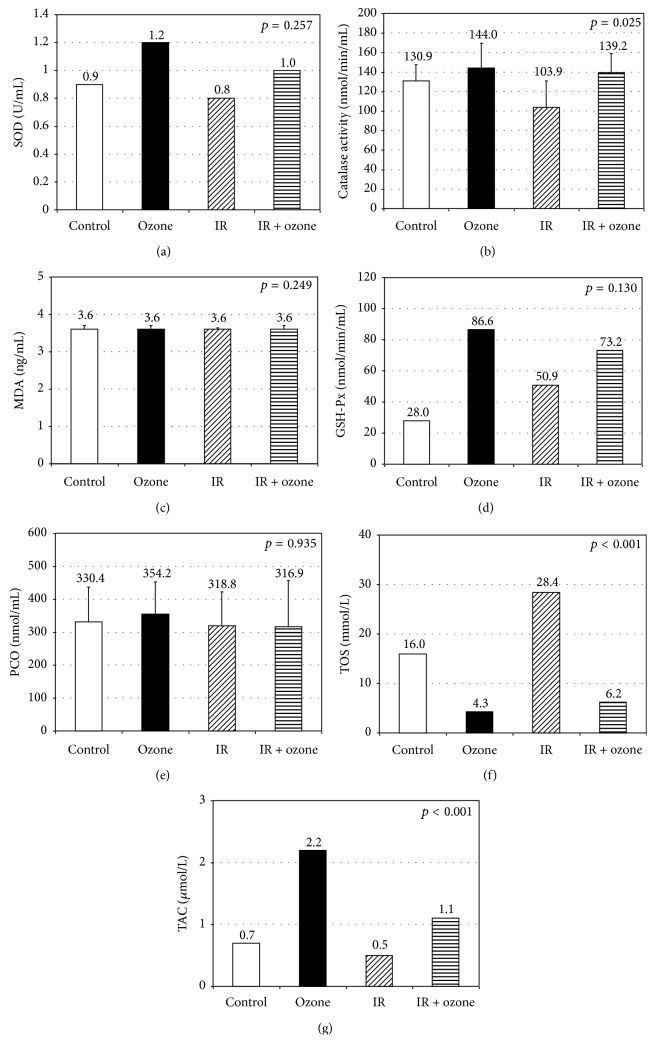
Biochemical measurements regarding the study groups. The white and black bars represent control and Ozone groups, respectively. The hatched bars represent the situation after IR injury (diagonal lines). The hatched bars represent the situation after Ozone exposure (horizontal lines) prior to intestinal IR injury.

In addition, SOD, CAT, MDA, GSH-Px, and PCO were evaluated in intestinal tissues; TOS and TAC were measured in blood samples. Although the difference between them was not significant, among the antioxidant parameters, the SOD, GSH-Px, and CAT values were found to be highest in the ozone group and lowest in the IR group. No difference was found between the groups in terms of MDA and PCO. Statistically significant difference was found between terms of TOS and TAC values (*p* < 0.001). In both TOS and TAC values, significant difference was found between ozone and IR group, control and ozone group, control and IR group, control and IR + ozone group, ozone and IR + ozone group and, and IR and IR + ozone group. The TOS parameter was found to be highest in the IR group and the TAC parameter was highest in the ozone group and lowest in the IR group (Figure 3).

## 4. Discussion

Intestinal IR injury usually occurs in critical patients and leads to systemic inflammation and multiple organ failure (MOF) [24]. Oxidative stress is the major mechanism that triggers IR injury. Advantageous effects of ozone as an antioxidant agent have been shown in several pathologies [25, 26]. Ozone maintains cellular antioxidant systems including glutathione, SOD, and enzymatic reactions, preparing the host to confront the pathophysiologic conditions mediated by oxidative stress with repetitive doses [27]. The dose and the way of administration are important for antioxidant efficiency of ozone. In our study we determined that ip application of ozone for 5 days showed a beneficial effect on both antioxidant and histopathological variables in intestinal IR injury, which is parallel to the findings of Kesik et al. [27]. Kesik et al. [27] administrated ozone at a dose of 0.72 mg/kg daily via i.p. route for 15 days. In this dose and repetition they reported an increase in SOD and GSH-Px activity and maintained MDA levels stable in metoteraksat induced intestine injury. No data were included concerning the effect of ozone in blood enzymes [26]. Simon et al. reported repeated ip application of ozone in low doses resulted from decrease in total antioxidant capacity in blood [26]. Plasma antioxidant status is the result of the interaction of many different compounds and systemic metabolic interactions. Recent reports indicate that the separate measurement of different oxidant and antioxidant molecules is impractical since there are a great number of oxidants and antioxidants in the body and their effects are additive [28]. Moreover total antioxidant capacity of a blood sample is needed to determine an effective and a nontoxic ozone dose [29]. In addition to antioxidant parameters such as SOD, CAT and GSH-Px, total oxidant status, and total antioxidant capacity (TOS and TAC values) were included in our study.

In IR injury, oxidative stress is followed by a cascade involving inflammatory cell activation, systemic inflammatory mediator production, increased bacterial translocation, the release of bacterial products such as endotoxin and leading to their systemic spread, systemic inflammatory reaction, and multiple organ failure [11]. In order to inhibit the activation of inflammatory reaction and to achieve an antioxidant activity both in tissue and blood level, we used ozone for 5 consecutive days at 1 mg/kg dose, which was reported as an inhibitory dose for TNF-*α* [30]. Ozone oxidative preconditioning results in an increase in SOD, GSH-Px values, which is parallel to our findings [27, 31]. Higher SOD, CAT, GSH-Px, and the TAC values in ozone group supports that both local and systemic antioxidant responses are induced by ozone preconditioning at the applied dose. Similar to our previous experience [20], the IR event resulted in an intestinal mucosal injury manifested by an increased Chiu's intestinal injury score. The TOS value measured in blood samples was highest in IR group and significantly lower in the Ozone + IR group compared to IR group. These TOS results were parallel with the histopathological results. Intestinal injury scores indicating injury to the jejunum and ileum were found to be highest in the IR group, while, in the Ozone + IR group, these scores were found to be one-fourth of those of the IR group. Intestinal injury score was decreased and antioxidant parameters were enhanced by ozone ip application. Ozone oxidative preconditioning appears to restore the oxidant balance, minimizing tissue injury caused by IR injury. The use of ozone in humans and animals is still considered controversial [32, 33]. For these reasons, ozone therapy has limited acceptance in clinical practice, and most of the studies on ozone therapy have been experimental. One limitation of our study is that our study shows the results of ozone administration before IR injury. Indeed, an important aspect of the topic is that ozone, being a strong oxidizer, can stimulate the increase of anti-oxidant systems, eventually inhibiting the evolution of oxidative stress. However it seems that the clinical use of the so called oxidative preconditioning phenomenon is rather limited. Our paper could have been significantly better if outcome measurements would have been determined in both pretreatment and posttreatment models. Although it may be thought that the beneficial effect of ozone in clinical use is not marked, it is our suggestion that its use especially prior to organ and tissue transplantation and intestine, lung, and liver resections, all of which are associated with ischemia reperfusion injury, will yield favorable results.

Other limitation is the lack of standardization of tissue MDA and PCO analysis results to tissue protein amount. This lack of standardization leads histopathological data to be consistent with blood analysis results, while they are not so with tissue analysis results. Recently the stimulatory effect of ozone in postconditioning has been reported [34, 35]. Treatment with ozone prevents intestinal mucosal damage, stimulates cell proliferation, and inhibits programmed cell death following intestinal IR in rat. Increased villus height and crypt depth were reported as a marker of increased absorptive surface area [35]. Our results regarding the villus height and crypt depth were parallel with the results of Haj et al. [35]. Higher villus height and crypt depth in the ozone + IR group compared to IR group suggests the increased cell production and accelerated migration along the villus in response to ozone preconditioning of IR injury.

The effects of ozone remain controversial; however, in many studies favourable effects of ozone on IR injury in various organs have been shown. In the present study ip application of ozone for five days resulted an increase in both separate and total antioxidant parameters. Ozone preconditioning has a positive effect on both antioxidant and histopathological variables in intestinal IR injury. Further studies are required so that this effect can be utilized in humans.

## Figures and Tables

**Table 1:** Evaluation of villus height and crypt depth in study groups.

Variables	Control	Ozone	IR	Ozone + IR
Jejunum villi	258.9 (75.5)^a,b^	278.3 (31.4)^c,d^	131.0 (41.3)^a,c,e^	205.8 (39.6)^b,d,e^
Jejunum crypts	117.3 (35.6)^a,b^	120.4 (10.2)^c,d^	67.3 (13.2)^a,c,e^	99.3 (10.4)^b,d,e^
Ileum villi	261.8 (190.2)^a,b^	246.8 (108.7)^c,d^	116.8 (54.4)^a,c^	142.0 (16.2)^b,d^
Ileum crypts	118.0 (17.4)^a,b^	110.6 (33.2)^c,d^	74.6 (39.0)^a,c^	70.9 (10.0)^b,d^

Data are expressed as mean. Rows that share the same letters (superscripts) are significantly different from each other (*p* < 0.001 for a, b, c, d, and *p* < 0.01 for e).

**Table 2:** The intestine injury scores for experimental groups.

Variables	Control	Ozone	IR	IR + Ozone
Jejunum	0 (0)^a,b^	0 (0)^c,d^	3 (1)^a,c,e^	1 (0.5)^b,d,e^
Ileum	0 (0)^a,b^	0 (0)^c,d^	4 (1)^a,c,e^	1 (0.5)^b,d,e^

Injury in intestinal mucosal tissues was graded semiquantitatively according to Chiu's classification. The mean intestinal injury grade of Ozone + IR group significantly decreased in both jejunum and ileum compared to IR group. Data are expressed as mean. Rows that share the same superscripts are significantly different from each other (*p* < 0.001 for a, b, c, d, and *p* < 0.01 for e).
